# Case Report: A Mirror Within: Open Appendectomy in a Patient With Situs Inversus Totalis and Acute Appendicitis

**DOI:** 10.1002/ccr3.70678

**Published:** 2025-08-04

**Authors:** Waleed Ahmad, Haadia Ali, Wasif Majeed Chaudhry, Javed Iqbal, Asraf Hussain

**Affiliations:** ^1^ Lahore Medical and Dental College Lahore Pakistan; ^2^ Ghurki Trust Teaching Hospital Lahore Lahore Pakistan; ^3^ Hamad Medical Corporation Doha Qatar; ^4^ Chitwan Medical College Bharatpur Nepal

**Keywords:** case report, left sided acute appendicitis, open appendectomy, situs inversus totalis, surgery

## Abstract

In case of situs inversus totalis, acute appendicitis can develop in the left lower quadrant, and delayed diagnosis and complications can occur. Clinicians should be highly suspicious and should use imaging to confirm diagnosis. Detection of anatomical differences leads to intervening steps at an initial stage and optimal surgical success in peculiarities.

## 1 Introduction

Acute appendicitis causes approximately 6% of emergency department cases that lead to acute abdominal pain [1]. The manifestation of left‐sided acute appendicitis is unusual, yet situs inversus and midgut malrotation exist as frequent congenital irregularities that result in this presentation. The appendix extending into the left lower quadrant area represents a potential cause of this condition [2]. The condition SIT displays as a relatively uncommon medical presentation which exchanges the typical orientation of internal organs to mirror‐like opposites of normal organization patterns. The condition exists in two forms: partial manifestation limited to the thoracic and abdominal areas, or full manifestation that reverses both abdominal and thoracic organs. Left‐sided acute appendicitis proves difficult to diagnose because its uncommon presentation tends to delay the correct diagnosis [3]. We describe an unusual case of SIT with left‐sided acute appendicitis that required open appendectomy as the treatment method.

## 2 Case History/Examination

The emergency department received a 16‐year‐old boy of Pakistani origin who experienced left iliac fossa abdominal pain that started 2 days prior. He experienced recurrent vomiting and anorexia along with nausea during the 2 days, while his fever manifested during the previous day. Prior to his pain onset in the umbilical area, the patient kept his usual health status before his symptoms spread to the left iliac fossa. The pain started as a gradual occurrence with intense severity and a dull quality. No specific triggers for discomfort emerged during medical treatment, even though painkillers slightly decreased the severity. The patient exhibited mild fever but showed no symptoms of chills or body rigors. The patient did not have any noteworthy past medical or surgical history.

The physical findings showed the abdomen to be scaphoid in shape and the umbilicus being central and inverted. No prominent pulsations or scar marks were visible. On superficial palpation, the patient had extensive abdominal pain along with deep tenderness being most severe in the left lower quadrant and hypogastric region. Rebound tenderness was also present. Fluid thrill and shifting dullness were negative. Bowel sounds were audible but sluggish. The rest of the systematic examinations were unremarkable.

## 3 Investigations/Differential Diagnosis/Treatment

**FIGURE 1:**
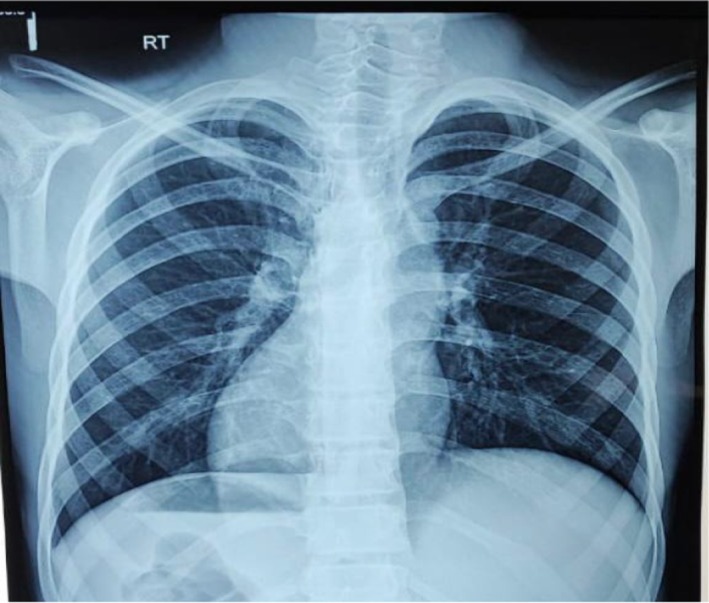
Chest X‐ray presents evidence of dextrocardia along with a gastric bubble located in the right abdominal region.

The clinical laboratory results indicated leukocytosis with a total white blood cell count measured at 11.47 × 10^9^/L while neutrophils formed 92% of all detected cells. Other laboratory tests as well as the urine analysis yielded results that fell in the standard range. The chest X‐ray showed dextrocardia as well as an elevated left hemidiaphragm and a right‐sided gastric air shadow suggesting inverted anatomical structures (Figure 1). A noncompressible blind‐ended tubular structure measuring 9.7 mm in diameter was noted within the left iliac fossa area through ultrasound imaging of the abdomen. The physical examination revealed probe‐induced tenderness that caused the tissue to shrink and expand back quickly and peri‐appendiceal and interloop fluid accumulation. The liver appeared visually on the left side of the image while the spleen appeared on the right in this exam outcome. The differentials for the left‐sided pain included diverticulitis, ureteric colic, Meckel's diverticulitis, incarcerated or strangulated hernia, bowel obstruction, regional enteritis, and psoas abscess. Left‐sided acute appendicitis was the strongest interpretation of these diagnostic results.

**FIGURE 2:**
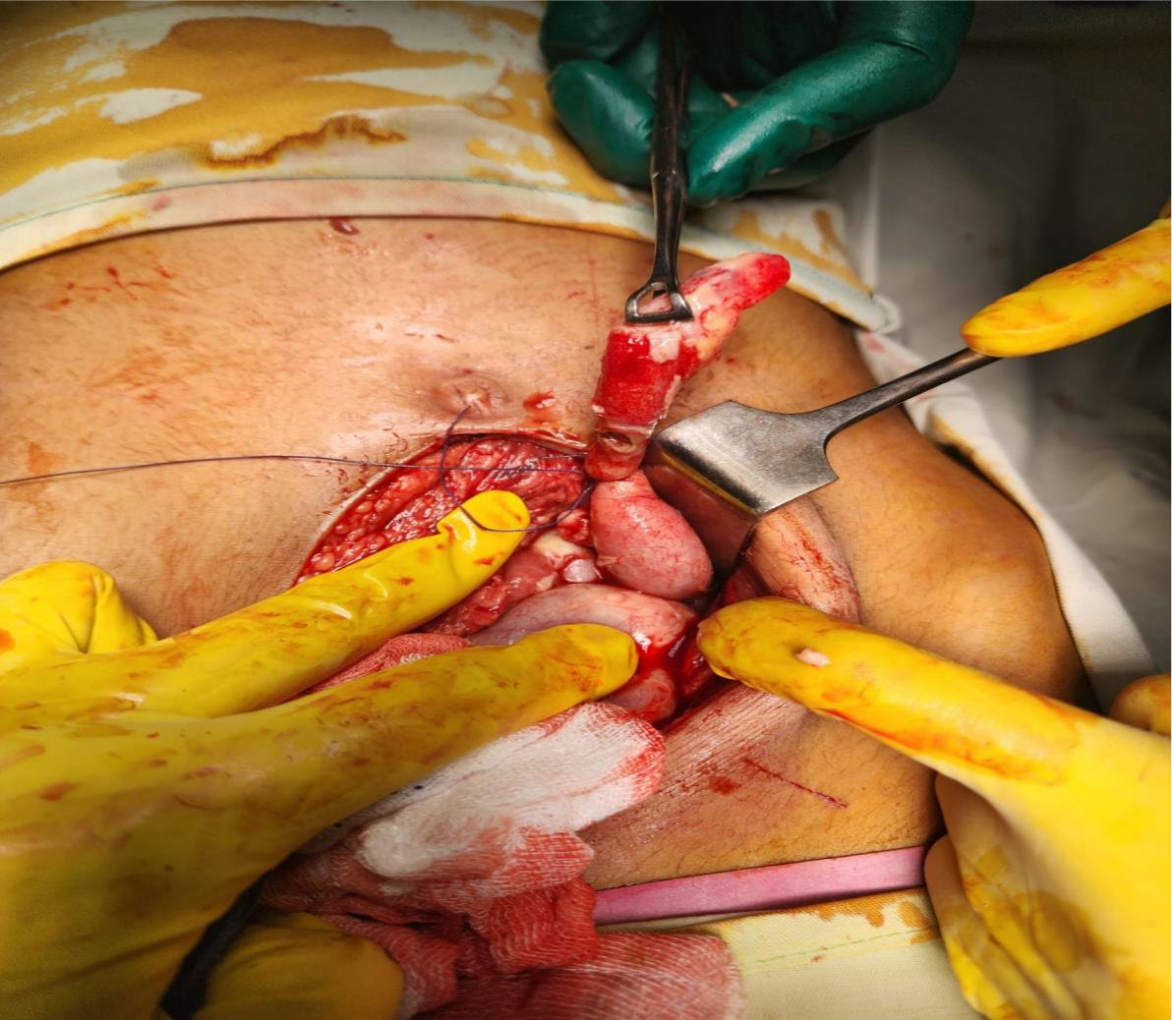
Intraoperative image presents a perforated appendix located in the left lower quadrant of the abdominal area.

The patient underwent an exploratory laparotomy through a midline incision, followed by an appendectomy. Intraoperatively, the appendix was found to be acutely inflamed with a perforation measuring approximately 1 cm just distal to the base, which itself appeared unremarkable (Figure 2). A fecolith was identified, and the appendix was adherent to the surrounding bowel and omentum. Approximately 50 mL of pus was drained from the left paracolic gutter and pelvis. A thorough intraoperative examination confirmed SIT, with the liver and gallbladder located on the left side, while the stomach and spleen were positioned on the right. The procedure was completed in approximately 44 min.

## 4 Conclusion and Results

The surgical drain together with the Foley catheter was removed on the second day after surgery. The patient experienced an unproblematic recovery, so was discharged on the third postoperative day. Histopathological analysis confirmed acute suppurative appendicitis. The patient showed complete recovery on his follow‐up visits.

## 5 Discussion

The position variability of the appendix leads to abdominal pain developing outside the right iliac fossa in about one‐third of acute appendicitis patients [4]. The manifestation of acute appendicitis pain in the left lower quadrant occurs infrequently [2]. The list of possible diagnoses for left iliac fossa pain in adults comprises bowel obstruction alongside acute sigmoid diverticulitis as well as strangulated or incarcerated hernia together with small bowel enteritis, which combines with Meckel's diverticulum, ruptured ovarian cyst, ectopic pregnancy, ureteric colic, acute epididymitis, alongside psoas abscess, and acute appendicitis [5, 6]. Left‐sided acute appendicitis can occur because the appendix extends beyond the normal right quadrant or when the cecum displays redundancies in its area [7]. Congenital anomalies such as midgut malrotation and SIT may also contribute to this rare presentation [3].

The intestinal failure to perform appropriate rotation around the superior mesenteric artery results in midgut malrotation affecting 0.03%–0.5% of newborns born alive [1]. This rare congenital condition of situs inversus exists in an autosomal recessive inheritance pattern and in it there is a 270° clockwise rotation of abdominal organs inside the embryo, thus fully transposing all visceral structures [8]. SIT affects approximately 0.001%–0.01% of people in the general population according to reports [2]. However, studies show this condition occurs in 0.016%–0.024% of patients who experience acute appendicitis. These patients experience delayed diagnosis and misdiagnosis of left‐sided acute appendicitis since their symptoms do not follow typical patterns. Despite the abnormal organ positioning, patients with SIT may still experience pain in the right lower quadrant, further complicating diagnosis [9].

A combination of clinical assessment, imaging, and diagnostic tests is essential for identifying left‐sided acute appendicitis. Diagnostic tools for such cases include physical examination combined with chest X‐ray along with ECG and ultrasound (USG), CT scan, and laparoscopy [4]. Right‐sided heart sounds together with left lower quadrant tenderness, left liver edge palpation, and testicular asymmetry with the right testis positioned below the left one make up the diagnostic assessment [2]. X‐ray examinations cannot identify acute appendicitis, but detecting dextrocardia together with right‐sided gastric bubble enables identification of SIT. Additionally, our patient displayed right axis deviation according to his ECG results.

The number of ultrasound (USG) and CT scans used for appendicitis diagnosis has grown substantially throughout recent years. The accuracy of ultrasound examinations can be reduced in individuals who are obese when bowel gas obscures the images or when the operator's experience determines the test's results [7]. CT scans remain the most effective tool for appendicitis diagnosis through their ability to show detailed images of abnormal appendix positioning with sensitivity rates reaching 90%–98% [4]. A definitive diagnosis established the patient's SIT along with left‐sided acute appendicitis by combining clinical presentations with examination results and chest X‐ray findings, which led to verification through abdominal ultrasound.

The medical care for patients diagnosed with SIT and left‐sided acute appendicitis follows conventional treatment methods as for standard appendectomy procedures [8]. Due to the likelihood of perforation, an exploratory laparotomy was performed for appendectomy. Intraoperative findings confirmed perforation with adhesions involving the omentum and bowel, which contributed to a slightly prolonged operative duration of 44 min compared to a standard open appendectomy.

## Author Contributions

**Waleed Ahmad:** conceptualization, writing – original draft. **Haadia Ali:** writing – original draft. **Wasif Majeed Chaudhry:** writing – original draft. **Javed Iqbal:** supervision, writing – review and editing. **Asraf Hussain:** validation, writing – review and editing.

## Ethics Statement

The Ethical Review Committee of Ghurki Trust Teaching Hospital Lahore granted approval to conduct this study with reference number 2025/02/R‐02.

## Consent

The patient granted permission via written consent for his anonymous details to be published in this research article.

## Conflicts of Interest

The authors declare no conflicts of interest.

## Data Availability

No new data is generated in this work.
